# Supplementary figures and images for: WHONDRS-GUI: a web application for global survey of surface water metabolites
Source: PeerJ. 2020 Jul 22;8:e9277. doi: 10.7717/peerj.9277 (PMC7382364; doi:10.7717/peerj.9277)

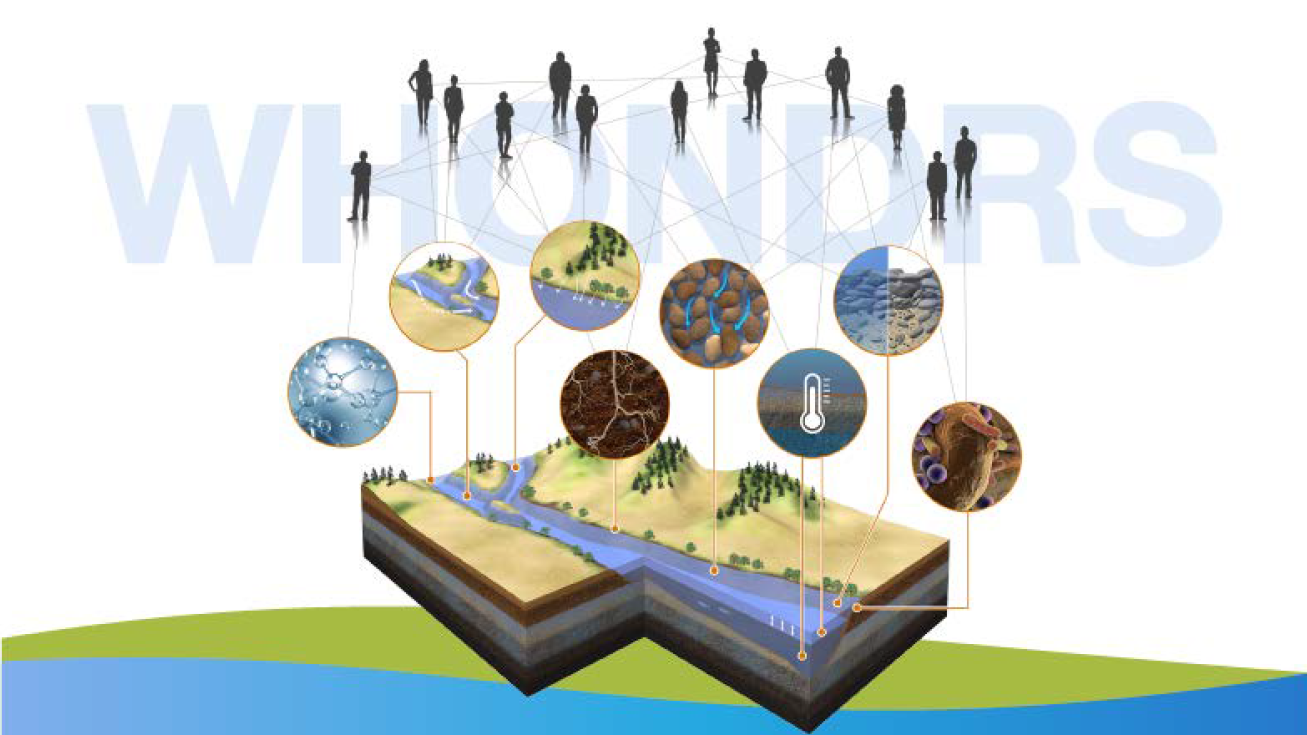

Supplement: Supplemental Information 1 [file peerj-08-9277-s001.zip › WHONDRS_S_Kit/www/bg.png]

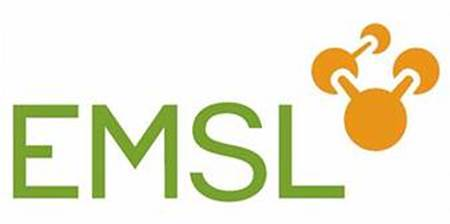

Supplement: Supplemental Information 1 [file peerj-08-9277-s001.zip › WHONDRS_S_Kit/www/emsl.png]

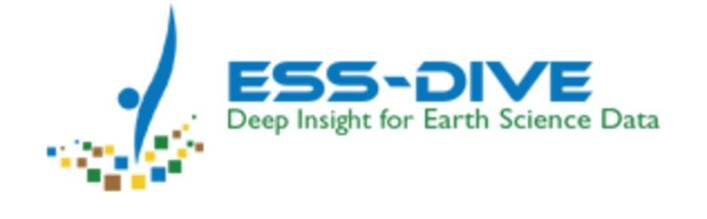

Supplement: Supplemental Information 1 [file peerj-08-9277-s001.zip › WHONDRS_S_Kit/www/ess.png]

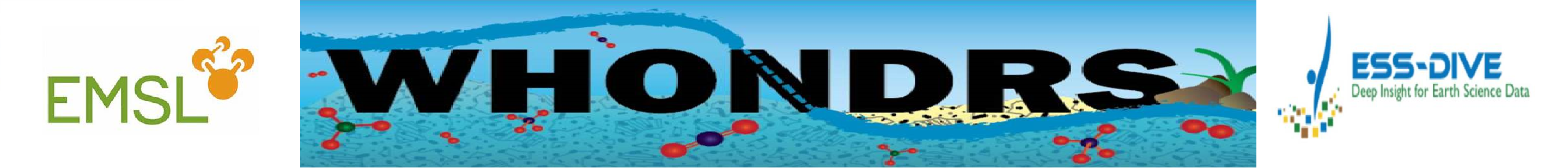

Supplement: Supplemental Information 1 [file peerj-08-9277-s001.zip › WHONDRS_S_Kit/www/header.png]

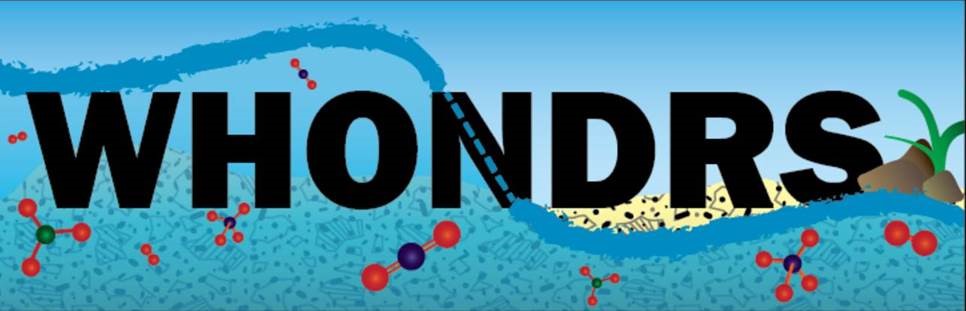

Supplement: Supplemental Information 1 [file peerj-08-9277-s001.zip › WHONDRS_S_Kit/www/whondrs.jpg]

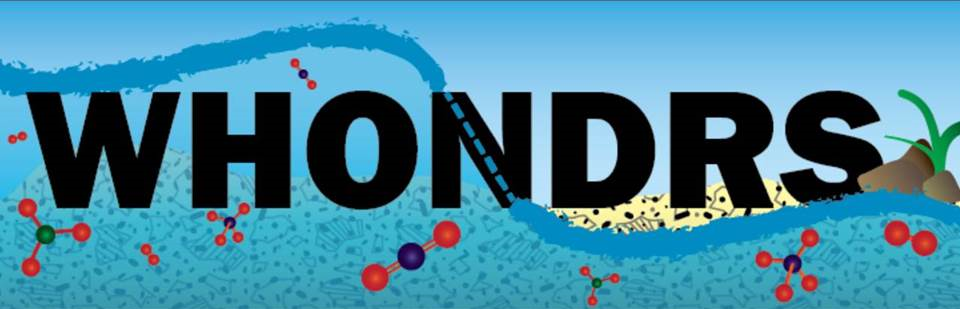

Supplement: Supplemental Information 1 [file peerj-08-9277-s001.zip › WHONDRS_S_Kit/www/whondrs.png]
